# Posterior lumbar interbody fusion for dysplastic lumbar spondylolisthesis with high-grade slippage in two adolescent siblings: two case reports

**DOI:** 10.1186/s13256-022-03534-0

**Published:** 2022-09-02

**Authors:** Masaki Tatsumura, Toru Funayama, Fumihiko Eto, Katsuya Nagashima, Yosuke Takeuchi, Masashi Yamazaki

**Affiliations:** 1 Department of Orthopaedic Surgery and Sports Medicine, Tsukuba University Hospital Mito Clinical Education and Training Center/Mito Kyodo General Hospital, 3-2-7 Miyamachi, Mito, Ibaraki 310-0015 Japan; 2 Department of Orthopaedic Surgery, Faculty of Medicine, University of Tsukuba, Tsukuba, Japan

**Keywords:** Dysplastic lumbar spondylolisthesis, Siblings, High-grade slip, Posterior interbody fusion, Cases report

## Abstract

**Background:**

Lumbar spondylolisthesis is reported to present with a familiar pattern, with the dysplastic type of spondylolysis being minor but more hereditary than the isthmic type. Siblings presenting during adolescence with neurological symptoms owing to high-grade dysplastic-type spondylolisthesis are rare.

**Case presentation:**

The older brother suffered from left leg pain and numbness and dysesthesia of the right posterior thigh and calf and could not walk without a crutch at the age of 15 years. He had canal stenosis with disc bulging and dysplastic bilateral facet joint at L5/S1. The L5 vertebral body was slipped anterior downward to S1, with a round-shaped S1 cranial endplate. We diagnosed dysplastic-type spondylolisthesis and performed posterior lumbar interbody fusion at L5/S with mild reduction and sublaminar wiring at L4/5. The younger brother had no neurological symptoms at age 14 years but suffered from bilateral lower leg numbness at age 18 years. He had canal stenosis with disc bulging at L4/5 and L5/S1 and dysplastic bilateral facet joint at L5/S1 with right pars defect. The L5 vertebral body was vertically displaced anterior to the S1 vertebral body, with an S1 round-shaped cranial endplate. We diagnosed dysplastic-type spondylolisthesis, and posterior lumbar interbody fusion at L4/5 and L5/S with reduction was performed. Their neurological symptoms of the lower legs disappeared, and interbody bone fusion was obtained.

**Conclusions:**

The surgical technique for high-grade dysplastic spondylolisthesis remains controversial in terms of in situ fusion versus reduction. We recommend that surgery be performed promptly at the end of bone maturation because neurological symptoms often appear at the end of bone maturation. Because high-grade slips are rare but siblings may be present, the sibling should also be screened when dysplastic spondylolisthesis is detected.

## Background

Malformed lumbar spondylolisthesis in the 5th lumbar (L5) vertebra causes low back and leg pain, and results in postural abnormalities such as pelvic retroversion and lumbar hyperlordosis. Imaging features include extension of the inferior articular process, trapezoidalization of L5, and anterior vertebral body slip with dome formation of the sacral endplate. Among the types of developmental spondylolistheses classified by Marchetti and Bartolozzi, the high dysplastic type is progressive and its natural course is poor [1]. On the other hand, it is rare for the patient to develop neurological symptoms with a high degree of slippage during the adolescent period. Surgery is necessary if there are neurological symptoms, but there is room for consideration regarding the timing of surgery and the need for reduction [2]. Hereditary involvement is suggested but not clear. We report herein the cases of two adolescent siblings treated by interbody fusion for dysplastic lumbar spondylolisthesis with high-grade slippage.

## Case reports

**Fig. 1:**
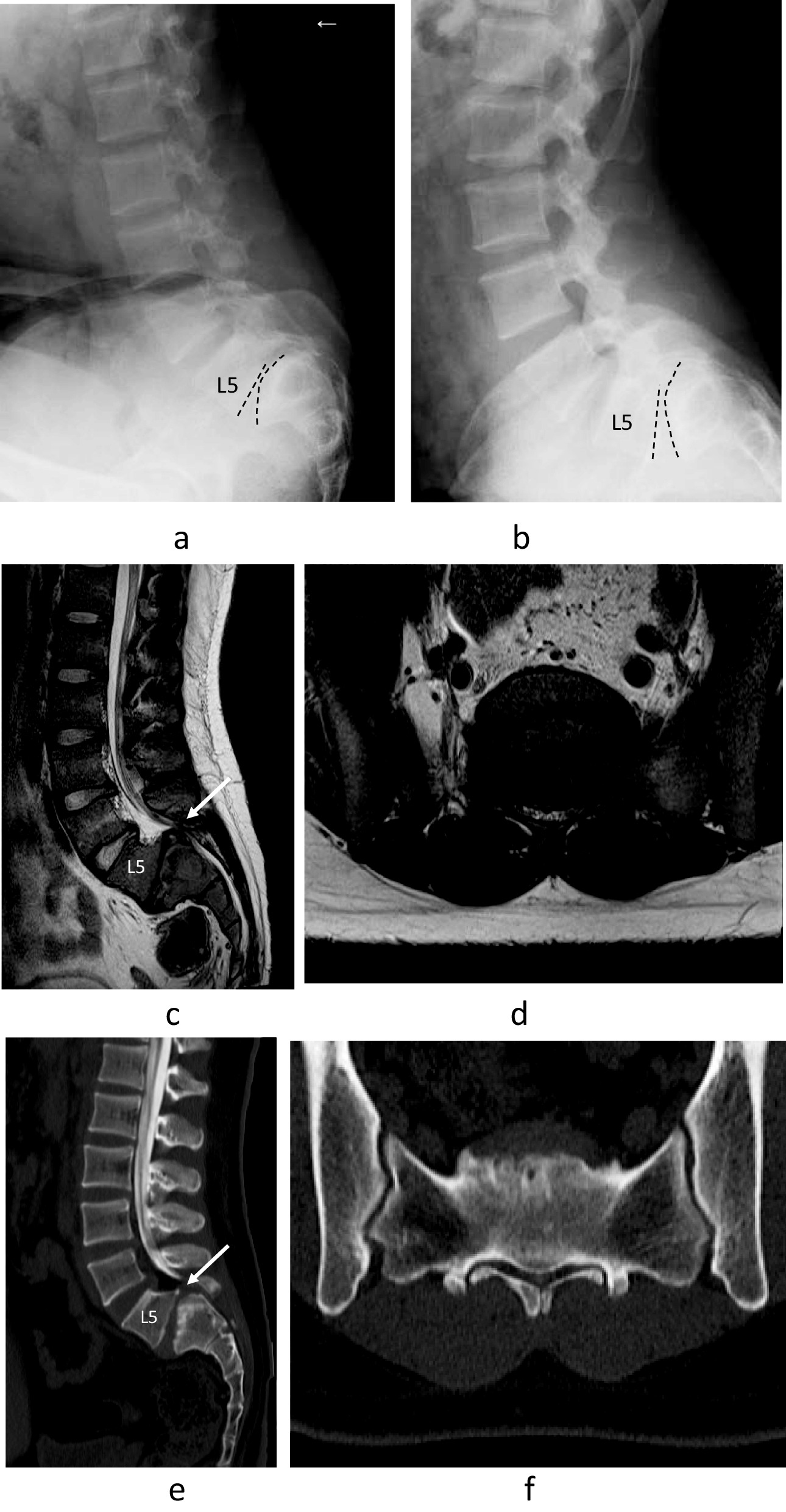
Lateral-view functional plain X-ray in the older brother showing slight instability between L5/S1 flexion and extension (**a**, **b**). Magnetic resonance images showing the L5 vertebra slipped anteriorly on S1 and the L5/S1 disc bulging apparent on the sagittal slice (arrow) (**c**). MRI also showed serious canal stenosis at L5/S1 with disc bulging apparent on the axial slice (**d**). Computed tomography with myelogram showed a vertically displaced anterior slip to S1, a round-shaped S1 cranial endplate and spinal canal stenosis (arrow) with dysplastic bilateral facets at L5/S1 (**e**, **f**)

**Fig. 2:**
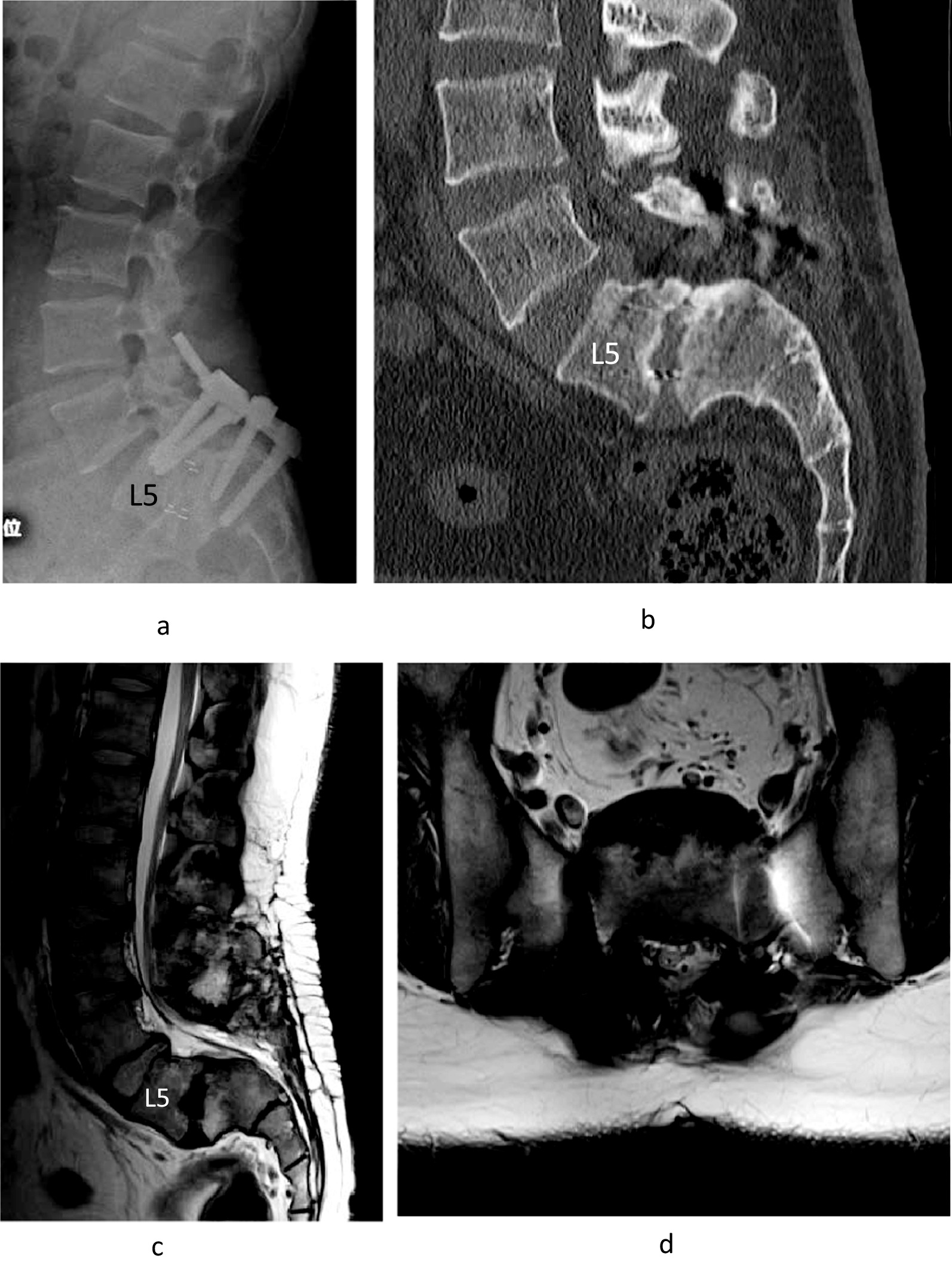
Posterior lumbar interbody fusion with mild reduction of L5/S1 was performed on the older brother with L5 and S1 pedicle screws (**a**). Computed tomography showed bony fusion between L5/S1 (**b**). MRI showed that decompression was established (**c**, **d**) 5 years after the operation

The older brother was a 15-year-old Japanese patient who presented with back pain and numbness in the left lower limb. One month after onset, he visited a previous doctor and was diagnosed with lumbar disc herniation. However, his left leg pain worsened seriously, and he came to our hospital and was diagnosed with dysplastic lumbar spondylolisthesis at 2 months after onset. He has no another medical history. There are no birth or developmental abnormalities, and this sibling is the only one with a family history related to spinal disease. Preoperatively, the patient was 171 cm tall and weighed 78 kg. There were no postural abnormalities, and it was difficult to determine lumbar spondylolisthesis from his external appearance. Neurological examination revealed left Achilles tendon reflex depression, negative bilateral straight leg raising (SLR) test, numbness and dysesthesia of the right posterior thigh and calf, and no bladder or rectal disorders. Preoperatively, scores on the Japan Orthopedic Association Low Back Pain Assessment Questionnaire (JOABPEQ) were 14 points for pain-related disorders, 75 points for lumbar dysfunction, 50 points for gait dysfunction, 30 points for social life disorders, and 89 points for psychological disorders. The preoperative visual analog scale of lower back pain and lower limb pain was 50 mm, and the degree of lower limb numbness was 20 mm. A functional lateral X-ray view indicated spondyloptosis at L5/S1 without instability (Fig. 1a, b). Magnetic resonance imaging (MRI) showed canal stenosis at L5/S1 with disc bulging (Fig. 1c, d). Computed tomography (CT) with myelogram revealed a downward slip at L5 anterior to the 1st sacral (S1) vertebral body with a round-shaped S1 cranial endplate (Fig. 1e). The spinal canal was narrow with a dysplastic bilateral facet at L5/S1 (Fig. 1f). Six months after onset, he had difficultly walking without a crutch, so it was decided to operate. Posterior lumbar interbody fusion (PLIF) with mild reduction at L5/S1 was performed with L5 and S1 pedicle screws and sublaminar wiring at the 4th lumbar (L4)/L5 with bilateral techmilon tapes between L5 and the S1 pedicle screw (Fig. 2a). Intraoperative transcranial motor-evoked potentials (Br-MEP) were used to prevent nerve damage during surgery. Because intraoperative Br-MEP showed amplitude decrease during reduction, we decided to make the reduction mild. We performed mild reduction, but mild motor weakness of the right gastrocnemius was observed after surgery. The motor paralysis of the right gastrocnemius muscle gradually improved, and the patient fully recovered after 1 year. We believe that the intraoperative Br-MEP would have avoided serious neurological damage. No neurological deficits remained 5 years after the operation. Postoperatively, the JOABPEQ indicated 100 points for pain-related disorders, lumbar dysfunction, gait dysfunction, social life disorders, and psychological disorders. The postoperative visual analog scale results for lower back pain, lower limb pain, and lower limb numbness were all 0 mm. CT revealed bony fusion between L5/S1 (Fig. 2b), and MRI indicated that decompression was established (Fig. 2c, d).

**Fig. 3:**
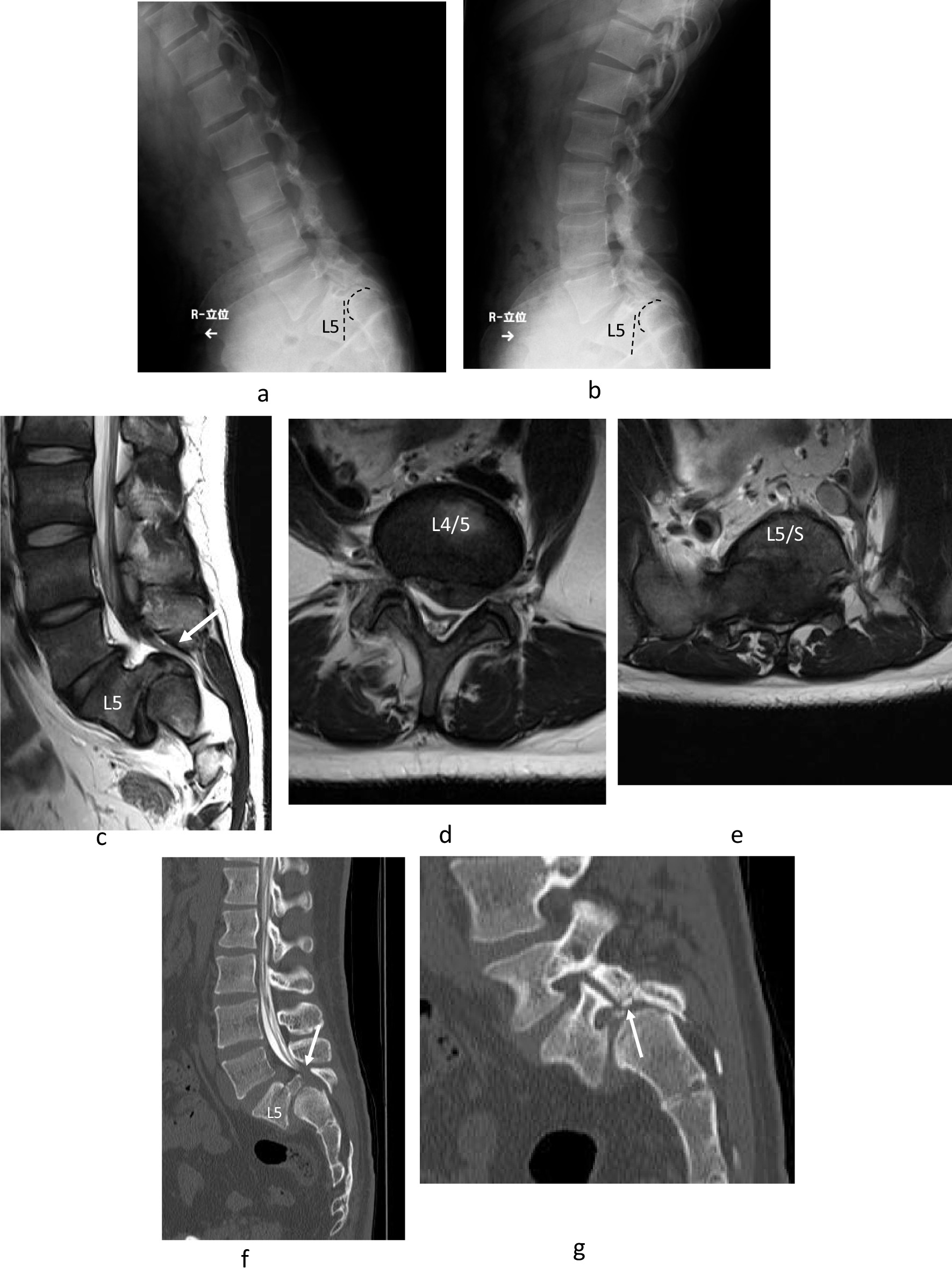
Lateral-view functional plain X-ray in the younger brother showing slight instability between L5 /S1 flexion and extension (**a**, **b**). MRI revealed that the L5 vertebra had slipped anteriorly on S1 and canal stenosis was present at L4/5 and L5/S1 with disc bulging (**c**–**e**). Computed tomography with myelogram showed a vertically displaced anterior slip to S1, a round-shaped S1 cranial endplate and spinal canal stenosis (arrow) with dysplastic bilateral facets at L5/S1 and unilateral pars defect of the right inferior articular process of L5 (arrows) (**f**, **g**)

**Fig. 4:**
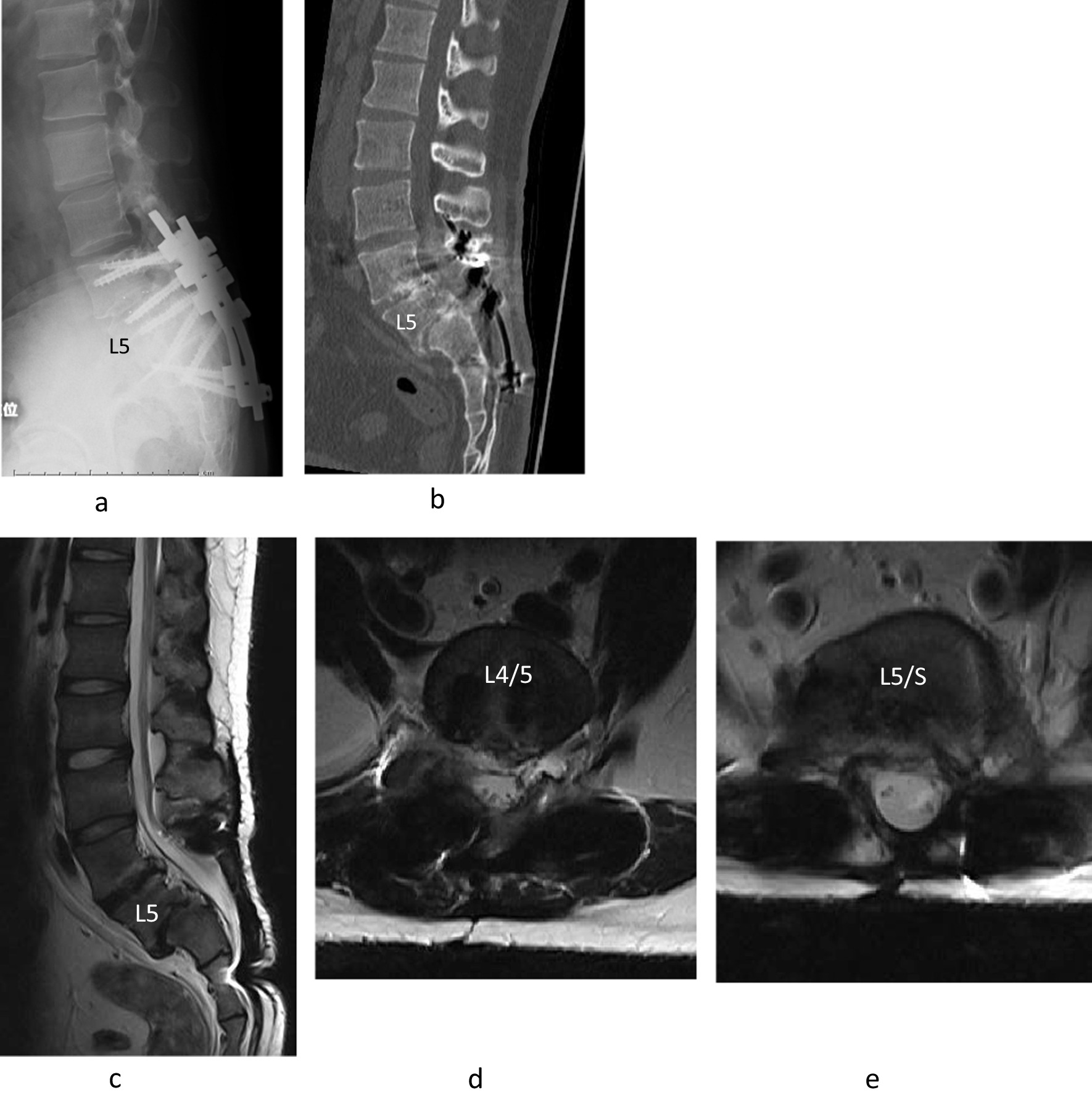
Posterior lumbar interbody fusion at L4/L5 and L5/S1 with reduction was performed on the younger brother with L4, L5, and S1 pedicle screws and S2 alar screws (**a**). Computed tomography indicated bony fusion between L5/S1 (**b**). MRI revealed that decompression was established (**c**–**e**) 5 years after surgery

The younger brother was a male Japanese patient who began to complain of low back pain at the age of 12 years. He came to the hospital after his older brother's surgery to investigate his spine and was diagnosed with dysplastic lumbar spondylolisthesis at the age of 14 years. He has no another medical history. There are no birth or developmental abnormalities, and this sibling is the only one with a family history related to spinal disease. As there were no neurological symptoms and his height was 161 cm tall and weighed 50 kg which were immature, it was decided to delay treatment but to follow up. At the age of 18 years, numbness in the bilateral lower limbs appeared. Preoperatively, the patient was 169 cm tall and weighed 76 kg, had no resting pain, no reflex of bilateral Achilles tendon, negative bilateral SLR, no dysesthesia, and no bladder or rectal disorders. There were no postural abnormalities, and it was difficult to determine lumbar spondylolisthesis from his external appearance. The JOABPEQ indicated 100 points for pain-related disorders, lumbar dysfunction, and gait dysfunction, 78 points for social life disorders, and 93 points for psychological disorders. The preoperative visual analog scale of lower back pain and lower limb pain was 0 mm, and the visual analog scale of lower limb numbness was 22 mm. A functional lateral X-ray view showed spondyloptosis at L5/S1 (Fig. 3a, b). MRI revealed canal stenosis at L4/5 and L5/S1 (Fig. 3c–e). By CT, the L5 vertebral body was vertically displaced anterior to the S1 vertebral body with an S1 round-shaped cranial endplate (Fig. 3f) and unilateral pars defect of the right inferior articular process of L5 (Fig. 3g). Neurological symptoms had worsened 2 months after he felt the numbness, so surgery was indicated. PLIF at L4/L5 and L5/S1 with reduction was performed with L4, L5, and S1 pedicle screws and S2 alar screws (Fig. 4a). Br-MEP was used to prevent nerve damage during surgery, and no deterioration of neurological symptoms occurred after surgery. Postoperatively, the JOABPEQ indicated 100 points for pain-related disorders, lumbar dysfunction, gait dysfunction, social life disorders, and psychological disorders. The postoperative visual analog scale results for lower back pain, lower limb pain, and lower limb numbness were all 0 mm. CT showed bone fusion between L4/5 and L5/S1 (Fig. 4b), and MRI revealed that decompression was obtained 2 years after surgery (Fig. 4c–e).

All procedures including the review of patient records used in this report were approved by the institutional review board (no. 21–28). Written informed consent was obtained from these patients for publication of these case reports and any accompanying images.

## Discussion

According to the Wiltse classification system [3], the two types of pediatric spondylolisthesis are isthmic and dysplastic. Marchetti and Bartolozzi subdivided spondylolisthesis into high dysplastic and low dysplastic [1]. Regarded as hereditary, previous epidemiological studies reported that 15% of the first-degree relatives of patients presented with isthmic-type lumbar spondylolisthesis but 33% presented with the dysplastic type [4]. Dysplastic spondylolisthesis is clearly different from isthmic spondylolisthesis in that it is accompanied by dysplastic facet joints and there is bony continuity of pars interarticularis. A plain X-ray or CT scan can be used to differentiate between those spondylolistheses. Albanese stated that dysplastic spondylolisthesis occurred one-third as often as isthmic spondylolisthesis [5]. The dysplastic type of spondylolysis is minor but is more hereditary than the isthmic type.

Despite the substantial debate regarding the genetic etiology of lumbar spondylolisthesis, there are only a few specific reports. There is a case report about highdysplastic developmental spondylolisthesis in two sets of identical twins in whom the imaging features were similar [6]. They had the same downward slip anterior to the S1 vertebral body with a round-shaped S1 cranial endplate, but not all morphological features were the same. The older brother was not scoliotic and did not have pars cleft, whereas the younger brother was scoliotic with a left side pars cleft. There also are two other reports of spondylolysis within a Japanese family [7, 8].

Dysplastic spondylolisthesis usually causes no symptoms in children; neurological symptoms usually begin in adolescence. As patients grow taller, their nerves are stretched, causing neurological symptoms. It is known that motor paralysis and leg pain due to neurological symptoms occurring during adolescence can make social life difficult. In some rare cases, surgery may be avoided by follow-up of high-grade spondylolisthesis [9], but these spondylolistheses often require surgical treatment because of neurological symptoms [10]. Failure to treat these cases surgically causes irreversible neurological symptoms. Surgery after the deformity has progressed excessively may increase the surgical invasiveness required for correction.

In situ posterolateral fusion is a well-known technique for which many surgeons have reported satisfactory clinical outcomes for low-grade spondylolisthesis [11]. On the other hand, the surgical technique for high-grade spondylolisthesis remains controversial in terms of in situ fusion versus reduction [12]. However, bone fusion may not be obtained without reduction in the case of high-grade slip because of the small bone contact area between the vertebral bodies. It has been reported that reduction causes radiculopathy. Even with high-grade spondylolisthesis, reduction should be performed only if the procedure is safe and does not worsen neurological symptoms. Reduction with Br-MEP has been useful to detect nerve damage during surgery [13]. The Br-MEP resulted in an alarm in our older patient, and we decided to change the degree of reduction to mild. The temporary paralysis of the lower extremity that occurred after surgery recovered spontaneously in that case. Because of significant reduction in the younger case, we felt it necessary to strengthen the immobilization and extended the fixation from L4 to S2.

There may be fewer choices for screw size in younger children who are almost asymptomatic. On the other hand, as neurological symptoms often appear at the end of bone maturation, we recommend that surgery be performed promptly at the end of bone maturation. Various surgical indications depend on the neurological condition or the degree of slippage. In the cases reported here, reduction with Br-MEP potentials has been useful to detect serious nerve damage during surgery. The results of surgery in two patients with dysplastic lumbar spondylolisthesis with high-grade slippage were favorable, with spontaneous recovery despite mild paralysis in one case.

## Conclusion

PLIF with reduction was performed for two adolescent siblings with dysplastic lumbar spondylolisthesis with high-grade slippage, and good results were obtained. Because high-grade slips are rare but siblings may be present, the sibling should also be screened when dysplastic spondylolisthesis is detected.

## Data Availability

The data analyzed in the current case are available from the corresponding author on reasonable request after anonymization so that the patient cannot be identified.

## Abbreviations

L5: The 5th lumbar
SLR: Straight leg raising
JOABPEQ: Japan Orthopedic Association Low Back Pain Assessment Questionnaire
MRI: Magnetic resonance imaging
CT: Computed tomography
S1: The 1st sacral
PLIF: Posterior lumbar interbody fusion
L4: The 4th lumbar
